# Geriatric nutritional risk index was associated with in-hospital mortality among cardiac intensive care unit patients

**DOI:** 10.3389/fnut.2023.1218738

**Published:** 2023-08-14

**Authors:** Yuefeng Li, Zhengdong Wang, Tienan Sun, Biyang Zhang, Xiangwen Liang

**Affiliations:** ^1^The First People’s Hospital of Yulin, Yulin, Guangxi, China; ^2^Department of Cardiology, Affiliated Anzhen Hospital, Capital Medical University, Beijing, China

**Keywords:** MIMIC-IV database, cardiac intensive care unit, geriatric nutritional risk index, nutritional status, in-hospital mortality

## Abstract

**Background:**

Identifying risk factors associated with cardiac intensive care unit (CICU) patients’ prognosis can help clinicians intervene earlier and thus improve their prognosis. The correlation between the geriatric nutrition risk index (GNRI), which reflects nutritional status, and in-hospital mortality among CICU patients has yet to be established.

**Method:**

The present study retrospectively enrolled 4,698 CICU patients. Based on the nutritional status, the participants were categorized into four groups. The primary endpoint was in-hospital mortality. The length of hospital stay and length of CICU stay were the secondary endpoints. To explore the correlation between nutritional status and in-hospital mortality, a logistic regression analysis was conducted. The nonlinear associations of GNRI with in-hospital mortality were evaluated using restricted cubic spline (RCS). Furthermore, subgroup analyses were conducted to evaluate the effect of the GNRI on in-hospital mortality across different subgroups, with calculation of the *p* for interaction.

**Result:**

A higher risk of malnutrition was significantly linked to an increased incidence of in-hospital mortality (High risk vs. No risk: 26.2% vs. 4.6%, *p* < 0.001), as well as a longer length of hospital stay (High risk vs. No risk: 15.7, 9.1–25.1 vs. 8.9, 6.9–12.9, *p* < 0.001) and CICU stay (High risk vs. No risk: 6.4, 3.8–11.9 vs. 3.2, 2.3–5.1, p < 0.001). An elevated GNRI was significantly associated with an increased risk of in-hospital mortality even after controlling for pertinent confounding factors (High risk vs. No risk: OR, 95% CI: 2.37, 1.67–3.37, *p* < 0.001, *p* for trend <0.001). Additionally, the RCS model showed a linear relationship between GNRI and in-hospital mortality, with the risk of in-hospital mortality significantly decreasing as GNRI increased (non-linear *p* = 0.596). Furthermore, in the subgroups of hypertension, ventricular arrhythmias, cardiac arrest, shock, and chronic kidney disease, there was a significant interaction between nutritional status and in-hospital mortality.

**Conclusion:**

Among CICU patients, a low GNRI was a significant predictor of in-hospital mortality. Furthermore, patients with a higher risk of malnutrition, as indicated by low GNRI values, experienced significantly longer hospital and CICU stays.

## 1. Introduction

Since its establishment in the 1960s with the objective of resuscitating patients with acute myocardial infarction (AMI), the coronary care unit (CCU) has undergone a transformation into a cardiac intensive care unit (CICU) (1–3). With the complexity of the clinical condition of patients, the current indications for CICU cover AMI, advanced heart failure (HF), cardiogenic shock (CS), organ failure, and multi-systemic critical illness (4). Patients admitted to the CICU often have many non-cardiac conditions in addition to cardiac disease, such as sepsis, acute renal failure, and acute respiratory failure (5, 6). These complications were associated not only with the severity of the underlying disease and the need for intensive care, but also with elevated morbidity and mortality rates, leading to greater resource utilization and medical costs (7–11). Therefore, identifying risk factors related to the prognosis of CICU patients is crucial for clinical physicians, which can help clinicians to intervene early in the treatment of patients and thus improve their prognosis.

Malnutrition is widespread in critically ill patients and is related to a worse prognosis (12–14). Calculated from serum albumin, height, and weight, the GNRI is a convenient and accessible indicator to evaluate the nutritional status of patients (15, 16). Patients with lower GNRI scores were considered to have poorer nutritional status and had worse outcomes (17, 18). The GNRI score is now used as a risk index for a variety of diseases, such as uremia, sepsis, and cardiovascular diseases (CVD) (19–21). Previous studies have linked GNRI to a poor outcome in various CVDs, including acute HF, coronary artery disease (CAD), and acute ST-segment elevation myocardial infarction (22–25). Hence, in critically ill patients admitted to the CICU, employing GNRI as a tool to assess nutritional status might enhance risk stratification, and providing timely nutritional support could potentially enhance long-term prognosis. However, no studies have been undertaken to investigate the impact of nutritional status on the prognosis of CICU patients. The aim of this study was to explore an association between GNRI and in-hospital mortality in CICU patients.

## 2. Methods

### 2.1. Population selection criteria

**Figure 1:**
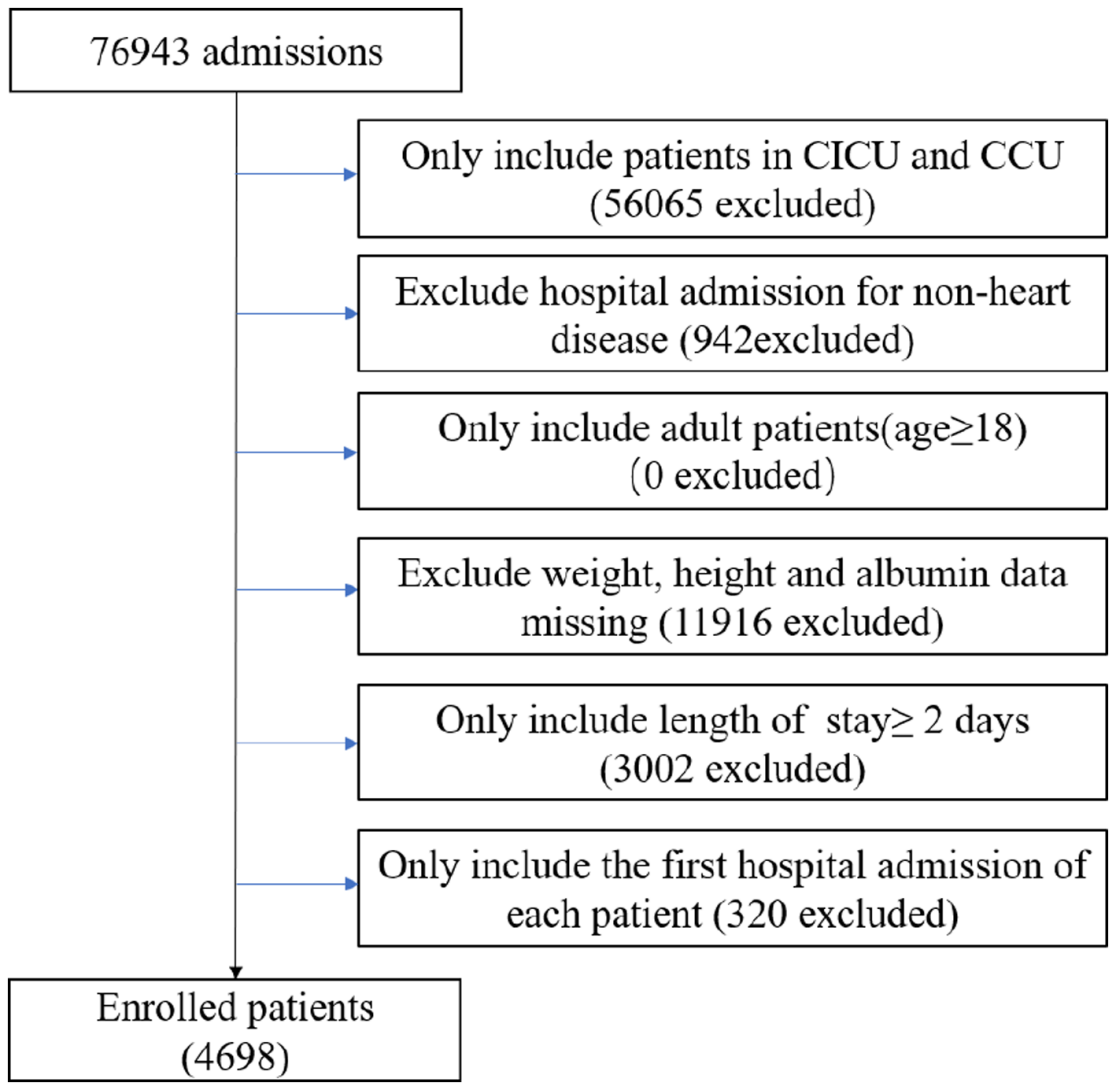
Flow chart of study population. CCU, coronary artery care unit; CICU, cardiac intensive care unit.

This was an observational, retrospective study that included patients from the CICU and CCU, extracted from the Medical Information Mart for Intensive Care IV (MIMIC-IV version 2.0). The database provides comprehensive and high-quality data on patients admitted to intensive care units at Beth Israel Deaconess Medical Center between 2008 and 2019 (26). As shown in Figure 1, all patients who were initially admitted to the hospital for a duration of more than two days were included. Patients with the following conditions were excluded: (1) non-cardiac hospitalization; (2) weight, height and albumin data missing; (3) age < 18 years. A total of 4,698 patients were enrolled.

### 2.2. Data extraction

The data utilized in this study was extracted from the publicly available critical care database known as MIMIC-IV (26). The following information was collected: demographics, vital sign, comorbidities and medical history, laboratory parameter and treatment (Details can be found in Supplementary material).

### 2.3. Definition of nutritional status and endpoints

According to GNRI, all patients were classified into four groups (15): No nutrition risk: GNRI≥98 (*n* = 1,560), Low nutrition risk: 92 ≤ GNRI <98 (*n* = 1,067), Moderate nutrition risk: 82 ≤ GNRI <92 (*n* = 1,214), High nutrition risk: GNRI<82 (*n* = 828). The GNRI index was calculated as follows: GNRI = [14.89 × serum albumin (g/dL)] + [41.7 × actual BMI/ideal BMI] (27). Ideal BMI was set to 22 kg/m^2^ (28). If the patient’s BMI exceeded the ideal BMI, the “actual BMI/ideal BMI” ratio was set to 1. The primary endpoint was in-hospital mortality. The secondary endpoints were length of hospital stay and length of CICU stay.

### 2.4. Statistical analysis

The baseline characteristics were reported as mean ± standard deviation (SD) for normally distributed quantitative data, median [interquartile range (IQR)] for skewed data, and number (%) for categorical data. Analysis of variance, Kruskal-Wallis, and chi-square tests were conducted to compare patient characteristics according to nutritional status. Binary logistic regression analysis was used to determine the association between nutritional status and in-hospital mortality, and the results were presented as odds ratios (OR) with corresponding 95% confidence intervals (CI). To account for relative confounding variables, a multivariate logistic analysis using the stepwise method with removal at *p* > 0.05 was performed on all baseline covariates listed in Table 1 (Details can be found in Supplementary material). Furthermore, we created a restricted cubic spline curve (RCS) based on the multivariate logistic regression model to investigate the relationship between GNRI and in-hospital mortality. Three knots were chosen for examination. In subgroup analysis, univariate binary logistic regression was used to assess the correlation between nutritional status and in-hospital mortality in various comorbidity subgroups. The results were expressed as OR and 95% CI, with *p* for interaction computed.

**Table 1:** Characteristics of patients stratified by nutritional status.

Characteristics	Total (*n* = 4,697)	Nutritional risk stratification				*p*-value
		No nutrition risk GNRI≥98 (*n* = 1,560)	Low nutrition risk 92 ≤ GNRI <98 (*n* = 1,067)	Moderate nutrition risk 82 ≤ GNRI<92 (*n* = 1,242)	High nutrition risk GNRI<82 (*n* = 828)	
Age(years)	68.4 ± 13.3	68.5 ± 12.7	69.7 ± 12.9	68.5 ± 13.4	66.2 ± 14.4	<0.001
Gender, *n* (%)						<0.001
Male	2,906 (61.9)	1,032 (66.2)	652 (61.1)	758 (61.0)	464 (56.0)	
Female	1791 (38.1)	528 (33.8)	415 (38.9)	484 (39.0)	364 (44.0)	
Race, *n* (%)						0.027
White	3,237 (68.9)	1,065 (68.3)	749 (70.2)	864 (69.6)	559 (67.5)	
Black	306 (6.5)	79 (5.1)	70 (6.6)	92 (7.4)	65 (7.9)	
Other	1,154 (24.6)	416 (26.7)	248 (23.2)	286 (23.0)	204 (24.6)	
Body mass index (kg/m^2^)	28.8 ± 6.8	29.4 ± 6.1	29.41 ± 6.93	28.48 ± 6.81	27.56 ± 7.52	<0.001
Systolic blood pressure (mmHg)	115.5 ± 21.8	115.0 ± 20.9	115.29 ± 21.36	116.11 ± 22.89	116.06 ± 22.34	0.487
Diastolic blood pressure (mmHg)	60.8 ± 15.0	60.2 ± 14.3	61.21 ± 15.32	61.30 ± 15.85	60.56 ± 14.80	0.201
Heart rate (beats/min)	84.7 ± 17.6	82.1 ± 14.9	84.00 ± 17.21	85.80 ± 18.11	88.47 ± 20.75	<0.001
*Comorbidities and medical history, n (%)*						
Congestive heart failure	2,609 (55.5)	726 (46.5)	671 (62.9)	772 (62.2)	440 (53.1)	<0.001
Coronary artery disease	3,296 (70.2)	1,168 (74.9)	782 (73.3)	872 (70.2)	474 (57.2)	<0.001
Acute myocardial infarction	1745 (37.2)	519 (33.3)	441 (41.3)	502 (40.4)	283 (34.2)	<0.001
Cardiomyopathy	411 (8.8)	111 (7.1)	109 (10.2)	132 (10.6)	59 (7.1)	0.001
Atrial fibrillation	2,830 (60.3)	895 (57.4)	650 (60.9)	791 (63.7)	494 (59.7)	0.008
Ventricular arrhythmias	701 (14.9)	170 (10.9)	161 (15.1)	222 (17.9)	148 (17.9)	<0.001
Atrioventricular block	453 (9.6)	157 (10.1)	106 (9.9)	120 (9.7)	70 (8.5)	0.623
Cardiac arrest	410 (8.7)	91 (5.8)	68 (6.4)	134 (10.8)	117 (14.1)	<0.001
Valve disease	2,162 (46.0)	833 (53.4)	514 (48.2)	539 (43.4)	276 (33.3)	<0.001
Shock	1,380 (29.4)	232 (14.9)	279 (26.1)	439 (35.3)	430 (51.9)	<0.001
Pulmonary embolism	191 (4.1)	36 (2.3)	34 (3.2)	58 (4.7)	63 (7.6)	<0.001
Endocarditis	152 (3.2)	9 (0.6)	22 (2.1)	50 (4.0)	71 (8.6)	<0.001
Dyslipidemia	2,778 (59.1)	1,059 (67.9)	659 (61.8)	701 (56.4)	359 (43.4)	<0.001
Hypertension	1924 (41.0)	779 (49.9)	435 (40.8)	422 (34.0)	288 (34.8)	<0.001
Diabetes	1810 (38.5)	572 (36.7)	429 (40.2)	495 (39.9)	314 (37.9)	0.203
Acute kidney injury	4,254 (90.6)	1,369 (87.8)	966 (90.5)	1,144 (92.1)	775 (93.6)	<0.001
Chronic kidney disease	1,500 (31.9)	392 (25.1)	363 (34.0)	487 (39.2)	258 (31.2)	<0.001
Malignancy	226 (4.8)	39 (2.5)	52 (4.9)	65 (5.2)	70 (8.5)	<0.001
*Laboratory parameters*						
White blood cell (10^9^/L)	10.64 ± 5.55	9.14 ± 4.37	10.01 ± 4.66	11.40 ± 5.63	13.13 ± 7.16	<0.001
Hemoglobin (g/dL)	11.01 ± 2.27	12.06 ± 2.17	11.15 ± 2.10	10.31 ± 2.03	9.92 ± 2.12	<0.001
Platelet (10^9^/L)	210.35 ± 96.79	207.80 ± 75.62	212.84 ± 90.18	209.94 ± 106.76	212.52 ± 121.66	0.526
ALT (U/L)	24 [16, 47]	23 [16, 36]	23 [15, 44]	26 [15, 64]	28 [16, 70]	<0.001
AST (U/L)	32 [21, 66]	26 [20, 40]	31 [21, 61]	40 [23, 93]	45 [25, 125]	<0.001
Creatinine (mg/dL)	1.58 ± 1.50	1.33 ± 1.02	1.56 ± 1.44	1.79 ± 1.84	1.77 ± 1.67	<0.001
Glucose (mg/dL)	142.40 ± 69.30	134.13 ± 54.98	142.38 ± 69.15	149.17 ± 80.63	147.85 ± 73.65	<0.001
Albumin (g/L)	3.43 ± 0.65	4.11 ± 0.26	3.57 ± 0.14	3.10 ± 0.20	2.43 ± 0.35	<0.001
Sodium (mmol/L)	138.21 ± 4.59	138.47 ± 3.65	138.12 ± 4.47	137.88 ± 5.06	138.31 ± 5.47	0.007
Potassium (mmol/L)	4.25 ± 0.65	4.22 ± 0.54	4.24 ± 0.67	4.30 ± 0.67	4.23 ± 0.76	0.012
*Treatment, n (%)*						
Oral anticoagulants	2,111 (44.9)	711 (45.6)	506 (47.4)	579 (46.6)	315 (38.0)	<0.001
Antiplatelet	4,213 (89.7)	1,504 (96.4)	969 (90.8)	1,096 (88.2)	644 (77.8)	<0.001
Beta-blockers	4,183 (89.1)	1,459 (93.5)	968 (90.7)	1,082 (87.1)	674 (81.4)	<0.001
ACEI/ARB	2,168 (46.2)	820 (52.6)	561 (52.6)	536 (43.2)	251 (30.3)	<0.001
Corticosteroids	1,412 (30.1)	393 (25.2)	337 (31.6)	390 (31.4)	292 (35.3)	<0.001
Vasoactive agent	3,613 (76.9)	1,228 (78.7)	823 (77.1)	920 (74.1)	642 (77.5)	0.033
Mechanical vent	3,241 (69.0)	1,065 (68.3)	700 (65.6)	832 (67.0)	644 (77.8)	<0.001
ECMO	69 (1.5)	10 (0.6)	11 (1.0)	17 (1.4)	31 (3.7)	<0.001

GNRI, geriatric nutrition risk index; ALT, alanine aminotransferase; AST, aspartate aminotransferase; ACEI, angiotensin-converting enzyme inhibitor; ARB, angiotensin receptor blocker; ECMO, extracorporeal membrane oxygenation.

All tests were two-sided, and statistical significance was defined as *p* < 0.05. R software was used to perform all data analysis.

## 3. Results

### 3.1. Patient characteristics

The patients were classified into four groups based on their nutritional status: No nutrition risk (*n* = 1,560), Low nutrition risk (*n* = 1,067), Moderate nutrition risk (*n* = 1,214), High nutrition risk (*n* = 828). Table 1 summarized the characteristics of the different nutritional states. Patients with high nutrition risk were younger, female sex, less often white, had a lower BMI but a higher heartrate, and were more likely to have a history of congestive HF, cardiomyopathy, atrial fibrillation, ventricular arrhythmias, acute myocardial infarction, cardiac arrest, pulmonary embolism, endocarditis, acute kidney injury, chronic kidney disease, shock and malignancy, but less often had coronary artery disease, valve disease, hypertension, and diabetes. Furthermore, patients with a high nutritional risk had higher levels of white blood cells, ALT, AST, creatinine, glucose, and potassium, while having lower levels of hemoglobin, sodium, and albumin. In addition, they received more corticosteroids, mechanical ventilation, and extracorporeal membrane oxygenation (ECMO), while receiving less oral anticoagulant, antiplatelet, beta-blocker, angiotensin-converting enzyme inhibitor/angiotensin receptor blocker (ACEI/ARB), and vasoactive agent therapy.

### 3.2. Association between nutritional status and adverse outcomes

Overall, in-hospital mortality rate was 12.2%. As nutrition risk groups increased, in-hospital mortality increased significantly (High risk vs. No risk: 26.2% vs. 4.6%, *p* < 0.001) (Table 2). Higher nutrition risk was significantly associated with the increased length of hospital stay (High risk vs. No risk: 15.7, 9.1–25.1 vs. 8.9, 6.9–12.9, *p* < 0.001) and CICU stay (High risk vs. No risk: 6.4, 3.8–11.9 vs. 3.2, 2.3–5.1, p < 0.001) respectively (Table 2). As shown in Table 3, in model 1, higher nutrition risk was associated with the increased risk of in-hospital mortality (High risk vs. No risk: OR, 95% CI: 7.45, 5.64–9.95, *p* < 0.001, *p* for trend <0.001). In Model 2, we adjusted for relevant confounding variables and found that a higher nutrition risk was significantly associated with an increased risk of in-hospital mortality (High risk vs. No risk: OR, 95% CI: 2.37, 1.67–3.37, p < 0.001, *p* for trend <0.001). When analyzing GNRI as a continuous variable, we found that an increase of one unit in GNRI was associated with a reduction in the risk of in-hospital mortality by approximately 0.07-fold in Model 1 and 0.04-fold in Model 2, respectively.

**Table 2:** Outcomes of patients stratified by nutritional status.

Outcomes	Total	Nutritional risk stratification				*p* value
		No nutrition risk GNRI≥98	Low nutrition risk 92 ≤ GNRI <98	Moderate nutrition risk 82 ≤ GNRI<92	High nutrition risk GNRI<82	
In-hospital mortality, *n* (%)	572 (12.2)	71 (4.6)	105 (9.8)	179 (14.4)	217 (26.2)	<0.001
Length of hospital stay (days)	10.9 [7.3, 17.0]	8.9 [6.9, 12.9]	10.8 [7.3, 15.6]	12.7 [8.3, 19.6]	15.7 [9.1, 25.1]	<0.001
Length of CICU stay (days)	4.1 [2.8, 7.1]	3.2 [2.3, 5.1]	4.0 [2.7, 6.2]	4.7 [3.1, 8.1]	6.4 [3.8, 11.9]	<0.001

Non-normally distributed continuous variables were presented as median (IQR). Categorical variables were presented as number (percentage). GNRI, geriatric nutrition risk index; CICU, cardiac intensive care unit.

**Table 3:** The association between nutritional status and in-hospital mortality.

Model	Logistic regression analysis		
	OR (95% CI)	*p* value	*p* for trend
Model 1			<0.001
No nutrition risk: GNRI≥98	Ref		
Low nutrition risk: 92 ≤ GNRI <98	2.29 [1.68, 3.14]	<0.001	
Moderate nutrition risk: 82 ≤ GNRI<92	3.53 [2.67, 4.73]	<0.001	
High nutrition risk: GNRI<82	7.45 [5.64, 9.95]	<0.001	
GNRI	0.93 [0.92, 0.94]	<0.001	
Model 2			<0.001
No nutrition risk: GNRI≥98	Ref		
Low nutrition risk: 92 ≤ GNRI <98	1.57 [1.09, 2.27]	0.016	
Moderate nutrition risk: 82 ≤ GNRI<92	1.65 [1.18, 2.33]	0.004	
High nutrition risk: GNRI<82	2.37 [1.67, 3.37]	<0.001	
GNRI	0.96 [0.97, 0.98]	<0.001	

Model 1: unadjusted. Model 2: adjusted for age, sex, race, white blood cell, sodium, congestive heart failure, coronary artery disease, atrial fibrillation, ventricular arrhythmias, cardiac arrest, shock, pulmonary embolism, dyslipidemia, diabetes, acute kidney injury, chronic kidney disease, oral anticoagulants, antiplatelet, beta-blockers, ACEI/ARB, corticosteroids, mechanical vent, ECMO. GNRI, geriatric nutrition risk index; ACEI, angiotensin-converting enzyme inhibitor; ARB, angiotensin receptor blocker; ECMO, extracorporeal membrane oxygenation; OR, odds ratio; CI, confidence interval.

**Figure 2:**
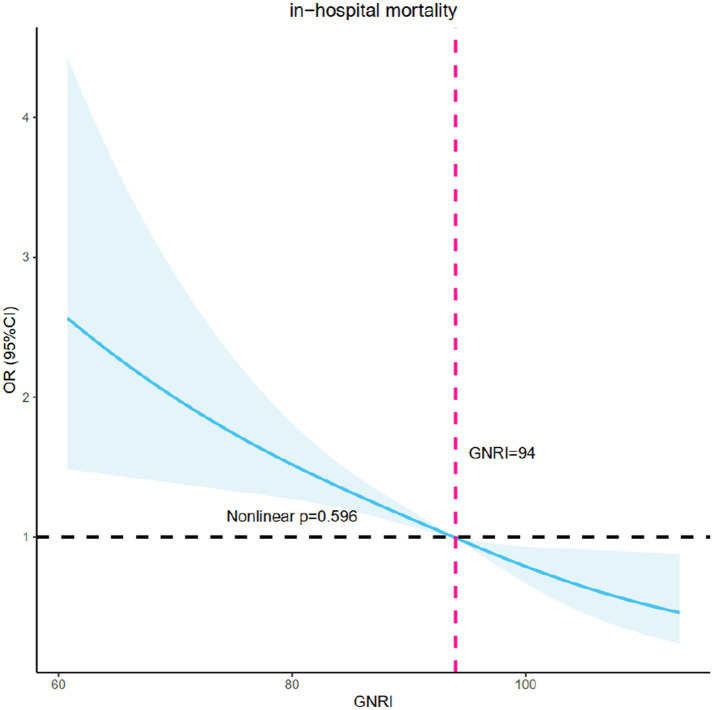
RCS model showing the association between the nutritional status and in-hospital mortality. RCS, restricted cubic spline curve.

Figure 2 displayed the use of restricted cubic splines (RCS) to visually represent the relationship between MACE and GNRI, as well as fit the model. After potential confounders were considered, a linear association between GNRI and in-hospital mortality was confirmed (non-linear *p* = 0.596). As GNRI increased, the risk of in-hospital mortality decreased significantly.

### 3.3. Subgroup analysis

In all subgroup analyses (Table 4), we found that patients with hypertension (*p* for interaction<0.001) had increased risks of in-hospital mortality for higher nutrition risk. But patients with ventricular arrhythmias (*p* for interaction = 0.046), cardiac arrest (*p* for interaction = 0.029), shock (*p* for interaction<0.001), and chronic kidney disease (*p* for interaction<0.001) had lower risks of in-hospital mortality. In the remaining subgroups, no significant interactions were found.

**Table 4:** Subgroup analysis of associations between in-hospital mortality and nutritional status.

Subgroups	*N*	No nutrition risk	Low nutrition risk	Moderate nutrition risk	High nutrition risk	*p* for interaction
Congestive heart failure						0.122
Yes	2,609	Reference	1.79 (1.23–2.61)	2.52 (1.77–3.57)	6.12 (4.29–8.71)	
No	2088	Reference	2.81 (1.59–4.97)	5.40 (3.27–8.93)	9.89 (6.07–16.12)	
Coronary artery disease						0.447
Yes	3,296	Reference	2.25 (1.57–3.23)	3.52 (2.52–4.90)	8.13 (5.80–11.41)	
No	1,401	Reference	2.40 (1.29–4.48)	3.61 (2.04–6.36)	6.76 (3.92–11.65)	
Acute myocardial infarction						0.625
Yes	1745	Reference	1.61 (1.02–2.55)	2.70 (1.78–4.10)	6.34(4.15–9.70)	
No	2,952	Reference	2.90 (1.90–4.45)	4.21 (2.84–6.25)	8.57(5.82–12.62)	
Cardiomyopathy						0.132
Yes	411	Reference	1.12 (0.47–2.67)	1.34 (0.60–3.00)	3.99 (1.73–9.19)	
No	4,286	Reference	2.49 (1.78–3.48)	3.96 (2.91–5.38)	8.08 (5.96–10.96)	
Atrial fibrillation						0.146
Yes	2,830	Reference	2.73 (1.87–3.98)	3.55(2.48–5.06)	7.10(4.96–10.17)	
No	1867	Reference	1.43 (0.81–2.55)	3.34(2.06–5.43)	8.00(5.03–12.72)	
Ventricular arrhythmias						0.046
Yes	701	Reference	1.40 (0.77–2.55)	2.37(1.39–4.02)	4.00(2.31–6.94)	
No	3,996	Reference	2.56 (1.77–3.71)	3.69 (2.61–5.22)	8.60 (6.13–12.06)	
Atrioventricular block						0.408
Yes	453	Reference	0.99 (0.39–2.50)	2.27 (1.06–4.89)	4.50 (2.04–9.92)	
No	4,244	Reference	2.56 (1.83–3.57)	3.79 (2.78–5.16)	8.05 (5.93–10.95)	
Cardiac arrest						0.029
Yes	410	Reference	1.36 (0.65–2.86)	2.48 (1.34–4.58)	3.25 (1.74–6.05)	
No	4,287	Reference	2.60 (1.83–3.70)	3.50 (2.51–4.88)	8.11 (5.84–11.24)	
Valve disease						0.532
Yes	2,162	Reference	2.25 (1.46–3.49)	3.02 (2.00–4.56)	6.84 (4.47–10.46)	
No	2,535	Reference	2.32 (1.49–3.63)	3.94 (2.63–5.90)	7.78 (5.23–11.56)	
Shock						<0.001
Yes	1,380	Reference	0.99 (0.66–1.48)	1.29(0.90–1.86)	1.86(1.30–2.65)	
No	3,317	Reference	4.62 (2.48–8.61)	6.10(3.35–11.12)	15.05(8.27–27.39)	
Pulmonary embolism						0.787
Yes	191	Reference	7.50 (0.85–65.99)	8.19 (1.01–66.45)	17.50 (2.24–136.71)	
No	4,506	Reference	2.20(1.60–3.02)	3.43(2.57–4.59)	7.16 (5.36–9.56)	
Endocarditis						0.468
Yes	152	Reference	1.26 (0.11–14.05)	0.89 (0.09–8.65)	2.52 (0.29–21.60)	
No	4,545	Reference	2.29 (1.67–3.14)	3.62 (2.71–4.83)	7.60 (5.69–10.14)	
Dyslipidemia						0.966
Yes	2,778	Reference	2.44 (1.62–3.68)	3.89 (2.66–5.69)	6.89 (4.62–10.29)	
No	1919	Reference	2.00 (1.23–3.22)	2.89 (1.87–4.46)	6.61 (4.35–10.05)	
Hypertension						<0.001
Yes	1924	Reference	3.33 (1.71–6.48)	10.68 (5.93–19.23)	16.89 (9.32–30.60)	
No	2,773	Reference	1.84 (1.29–2.63)	1.97 (1.41–2.75)	4.84 (3.48–6.73)	
Diabetes						0.308
Yes	1810	Reference	2.01 (1.26–3.20)	2.92 (1.90–4.48)	6.26 (4.07–9.62)	
No	2,887	Reference	2.50 (1.64–3.81)	4.04 (2.75–5.94)	8.46 (5.79–12.38)	
Acute kidney injury						0.305
Yes	4,254	Reference	2.19 (1.60–3.01)	3.40 (2.54–4.53)	6.94 (5.21–9.26)	
No	443	Reference	5.82 (0.60–56.65)	3.96 (0.35–44.20)	24.26 (2.85–206.35)	
Chronic kidney disease						<0.001
Yes	1,500	Reference	1.75 (1.12–2.72)	1.53 (1.00–2.34)	3.86 (2.50–5.95)	
No	3,197	Reference	2.50 (1.59–3.90)	5.81 (3.91–8.63)	11.17 (7.56–16.50)	
Malignancy						0.381
Yes	226	Reference	1.14 (0.30–4.36)	1.23 (034–4.38)	3.50 (1.10–11.13)	
No	4,471	Reference	2.35 (1.70–3.23)	3.69 (2.75–4.95)	7.62 (5.68–10.22)	

Binary logistic regression analysis was used and results were presented as OR (odds ratio) and 95% CI (confidence interval). *p* for interaction was calculated using binary logistic analysis to determine whether there is interaction between different subgroups and nutritional status.

## 4. Discussion

Our findings revealed that GNRI was an independent predictor of in-hospital mortality among CICU patients. The RCS analysis further confirmed a linear relationship between GNRI and in-hospital mortality. Furthermore, we found that higher nutrition risk was significantly related to the increased length of hospital stay and CICU stay. Significant interactions were observed in the relationship between GNRI and in-hospital mortality in hypertension, ventricular arrhythmias, cardiac arrest, shock, and chronic kidney disease subgroups.

Malnutrition, a condition characterized by an imbalance between the body’s energy intake and demands, has been unequivocally linked to cardiovascular disease (29). However, the underlying mechanism responsible for this association was multifaceted, with inflammation, metabolism, and aging all implicated in this pathological relationship (30, 31). Indeed, previous investigations have demonstrated that malnutrition was intricately linked to inflammation (30, 32). The inflammatory reaction, in turn, could antagonize albumin synthesis, a key protein involved in maintaining optimal nutritional status, and further aggravate malnutrition, engendering a self-perpetuating cycle of deleterious consequences (33). Furthermore, emerging evidence has suggested that malnutrition could precipitate the onset of various pathologies, such as free radical damage, impaired insulin secretion, lipolysis, and lipid oxidation. These adverse events, in turn, could incite tissue damage, diabetes, and fatty liver disease, thus perpetuating the vicious cycle of malnutrition (34–36). Importantly, previous research has also highlighted the unfavorable prognostic implications of malnutrition, manifesting as an adverse prognosis in various diseases, such as HF, CAD, and peripheral arterial disease (37–40).

Various systems are commonly employed in clinical practice to assess nutritional status, including subjective global assessment (SGA) (41) and mini-nutritional assessment (MNA) (42, 43). Nonetheless, many of these indicators have been discarded due to their complexity and vulnerability to subjective influences (41–43). Meanwhile, laboratory indices such as albumin (44) and hemoglobin (45) have been utilized to assess nutritional status and their association with patient prognosis has been established. However, these indicators are limited in that they only reflect a singular aspect and their predictive ability can be influenced by external factors. In recent years, GNRI has gained popularity as a commonly used tool in clinical nutrition assessment, primarily due to its convenience and accessibility (46). Moreover, it has been clinically established that a correlation between GNRI and the development and prognosis of several cardiovascular diseases, including HF, CAD, and stroke (47–49). A study that enrolled 2,299 patients with non-ST-segment elevation acute coronary syndrome found that a lower GNRI was significantly related to poor prognosis (50). An observational study showed that patients undergoing coronary artery bypass grafting with decreased GNRI had an increased incidence of MACE and a lower survival rate during long-term follow-up (51). According to a meta-analysis, low baseline GNRI was identified as a reliable predictor of cardiovascular events in CAD patients. In addition, another study conducted on elderly patients with HF demonstrated that a lower GNRI could independently predict MACE, thereby affirming the risk stratification ability of GNRI (22).

In the realm of scoring systems, GNRI exerts its preeminence by virtue of its remarkable faculty for risk stratification. The singularity of GNRI lies not only in its robustness, but also in its simplicity, which sets it apart from more intricate scoring mechanisms (52). As far as we knew, this study was the first to examine the correlation between GNRI and in-hospital mortality among CICU patients. As with prior research, the GNRI has been shown to be a reliable predictor of in-hospital mortality among CICU patients. This discovery reinforced the use of GNRI as a prognostic indicator in clinical settings and enhanced risk assessment and stratification based on traditional risk factors. Notably, among patients without ventricular arrhythmias, shock, chronic kidney disease or cardiac arrest, the effect of nutritional status on in-hospital mortality was enhanced, implying that clinicians should not ignore CICU patients without diseases that had a high case fatality rate, as paying attention to nutritional status and intervening accordingly could benefit patients more.

The RCS curve revealed a linear negative relationship between GNRI and in-hospital mortality: as nutritional status improved as measured by GNRI, the in-hospital mortality risk decreased, suggesting that clinicians might be able to improve poor outcomes by increasing GNRI with more aggressive treatment and better care. Furthermore, as the level of nutrition risk increased, the length of hospitalization and CICU stay rose significantly, compounding the emotional, physical, and financial stress experienced by patients. The potential explanation for this phenomenon was that patients with optimal nutritional status exhibited a more rapid convalescence from the ailment, thereby resulting in expedited hospital discharge and diminished expenses associated with hospitalization. As a result, indicators like the GNRI, which is more cost-effective and accessible, should receive more attention. When a full assessment of a patient’s health status is not possible in an emergency, the use of GNRI could quickly identify high-risk patients and provide clinicians with new treatment suggestions. This is especially true in medical settings that are deprived of adequate resources and infrastructure, such as those in geographically isolated regions or areas with poor healthcare facilities. Taken together, we believe that for patients with comorbid malnutrition in the CICU, the earlier their nutritional status is improved, the better their prognosis is likely to be.

While this study had some limitations. (1) This study only assessed the initial GNRI of CICU patients and did not record and analyze the dynamic changes in GNRI. (2) The use of public databases limited the collection of relevant information that could have influenced the model, such as detailed causes of death, left ventricular ejection fraction, specific coronary artery lesions, revascularization, types of myocardial infarction, and precise clinical symptoms. (3) Due to the retrospective nature of our study, we were unable to determine a specific cause for hospitalization. (4) Since it was a single-center retrospective study, it was susceptible to certain biases that might compromise the accuracy of the findings, thereby reducing their strength and rendering them incapable of establishing causality. Multi-central research is needed to further verify the current discovery among a wider range of people.

## 5. Conclusion

GNRI, being a simple and easily measurable tool in clinical practice, contributed significantly to the prognosis of in-hospital mortality among patients admitted to the CICU. Moreover, we found that higher nutrition risk, as indicated by low GNRI values, was significantly associated with prolonged hospital and CICU stays. Prospective, randomized studies are needed to establish whether interventions aimed at improving nutritional status could improve clinical outcomes. Moreover, we observed that higher nutrition risk, as indicated by low GNRI values, was significantly associated with prolonged hospital and CICU stays.

## Data availability statement

Publicly available datasets were analyzed in this study. This data can be found at: https://doi.org/10.13026/6mm1-ek67.

## Ethics statement

Written informed consent was obtained from the individual(s) for the publication of any potentially identifiable images or data included in this article.

## Author contributions

YL and ZW: conceptualization. TS and BZ: methodology. YL and ZW: writing – original draft. XL: writing – review and editing. All authors contributed to the article and approved the submitted version.

## Conflict of interest

The authors declare that the research was conducted in the absence of any commercial or financial relationships that could be construed as a potential conflict of interest.

## Publisher’s note

All claims expressed in this article are solely those of the authors and do not necessarily represent those of their affiliated organizations, or those of the publisher, the editors and the reviewers. Any product that may be evaluated in this article, or claim that may be made by its manufacturer, is not guaranteed or endorsed by the publisher.
