# Supplementary material for: Geriatric nutritional risk index was associated with in-hospital mortality among cardiac intensive care unit patients
Source: Front Nutr. 2023 Aug 14;10:1218738. doi: 10.3389/fnut.2023.1218738 (PMC10462258; doi:10.3389/fnut.2023.1218738)
Supplement: Supplementary file 1 [file Data_Sheet_1.docx]

**Data extraction.**

Following data were collected: demographics (age, sex, race), vital signs (systolic blood pressure, diastolic blood pressure, heart rate, BMI), diagnoses and comorbidities (congestive heart failure, coronary artery disease, acute myocardial infarction, cardiomyopathy, atrial fibrillation, ventricular arrhythmias, atrioventricular block, cardiac arrest, valve disease, shock, pulmonary embolism, endocarditis, dyslipidemia, hypertension, diabetes, acute kidney injury, chronic kidney disease, malignancy), laboratory parameter (white blood cell, hemoglobin, platelet, ALT, AST, creatinine, glucose, albumin, sodium, potassium), treatment(oral anticoagulant, antiplatelet, beta-blockers, ACEI/ARB, corticosteroids, vasoactive agent, mechanical ventilation, ECMO).

Model 1: Model 1 was unadjusted.

Model 2: Model 2 was adjusted for age, sex, race, white blood cell, sodium, congestive HF, CAD, atrial fibrillation, ventricular arrhythmias, cardiac arrest, shock, pulmonary embolism, dyslipidemia, diabetes, acute kidney injury, chronic kidney disease, oral anticoagulants, antiplatelet, beta-blockers, ACEI/ARB, corticosteroids, mechanical vent, ECMO.

Abbreviation: BMI: body mass index; ALT: alanine aminotransferase; AST: aspartate aminotransferase; ACEI: angiotensin-converting enzyme inhibitor; ARB: angiotensin receptor blocker; ECMO: extracorporeal membrane oxygenation.
